# Concrete Lessons: Policies and Practices Affecting the Impact of COVID-19 for Urban Indigenous Communities in the United States and Canada

**DOI:** 10.3389/fsoc.2021.612029

**Published:** 2021-04-23

**Authors:** Heather A. Howard-Bobiwash, Jennie R. Joe, Susan Lobo

**Affiliations:** ^1^ Department of Anthropology, Michigan State University, East Lansing, MI, United States; ^2^ Centre for Indigenous Studies, University of Toronto, Toronto, ON, Canada; ^3^ Department of Family and Community Medicine, College of Medicine, University of Arizona, Tucson, AZ, United States; ^4^ American Indian Studies, University of Arizona, Tucson, AZ, United States

**Keywords:** COVID-19, urban indigenous, human rights, indigenous rights, Canada, United States

## Abstract

Throughout the Americas, most Indigenous people move through urban areas and make their homes in cities. Yet, the specific issues and concerns facing Indigenous people in cities, and the positive protective factors their vibrant urban communities generate are often overlooked and poorly understood. This has been particularly so under COVID-19 pandemic conditions. In the spring of 2020, the United Nations High Commissioner Special Rapporteur on the Rights of Indigenous Peoples called for information on the impacts of COVID-19 for Indigenous peoples. We took that opportunity to provide a response focused on urban Indigenous communities in the United States and Canada. Here, we expand on that response and Indigenous and human rights lens to review policies and practices impacting the experience of COVID-19 for urban Indigenous communities. Our analysis integrates a discussion of historical and ongoing settler colonialism, and the strengths of Indigenous community-building, as these shape the urban Indigenous experience with COVID-19. Mindful of the United Nations Declaration on the Rights of Indigenous Peoples, we highlight the perspectives of Indigenous organizations which are the lifeline of urban Indigenous communities, focusing on challenges that miscounting poses to data collection and information sharing, and the exacerbation of intersectional discrimination and human rights infringements specific to the urban context. We include Indigenous critiques of the implications of structural oppressions exposed by COVID-19, and the resulting recommendations which have emerged from Indigenous urban adaptations to lockdown isolation, the provision of safety, and delivery of services grounded in Indigenous initiatives and traditional practices.

## Introduction

The deadly Coronavirus 2019 (COVID-19) pandemic laid bare gaps in national and local public health care policies, including missing data, that neglect specific issues and concerns facing Indigenous peoples in cities in both the United States (US) and Canada. Moreover, the positive protective factors generated in vibrant urban Indigenous communities are very often overlooked and poorly understood. As we show, the urban Indigenous experience with COVID-19 is shaped by historical and ongoing settler colonial actions which violate Indigenous human rights and are sustained through intersectional forms of systemic oppression and discrimination which exacerbate mistrust. We highlight Indigenous organizations, which are the lifeline of urban Indigenous communities, as they face challenges posed by key agencies that ignore or miscount their service populations, and the lessons provided as they adapted to lockdown isolation, the provision of safety, and delivery of services.

The rights of Indigenous peoples, as defined within the International Covenant on Economic, Social and Cultural Rights and the United Nations Declaration on the Rights of Indigenous Peoples, are inalienable and should not be diminished or extinguished when Indigenous peoples move to and through urban space (Belanger, 2011). Previously, we answered a special call from the United Nations Human Rights High Commission Special Rapporteur on the rights of Indigenous peoples, Francisco Calí Tzay, for his report to the United Nation’s 75th General Assembly (RISIU, 2020; UN General Assembly, 2020; UNHROHC, 2020). Ours was part of the response of the hemispheric-wide *Red de Investigaciones Sobre Indígenas Urbanos* (hereafter RISIU; Research Network on Urban Indigenous Peoples). The network includes several dozen academics, Indigenous and social movement leaders, and representatives of national organizations. The RISIU assembled information on the impacts of COVID-19 for urban Indigenous communities in nineteen countries of the Americas and was one of over 150 submissions received. In his UN report, the Special Rapporteur confirmed that Indigenous peoples were “the most harshly affected” by COVID-19 even though they only represent 6% of the world population because, Indigenous societies, already facing numerous existential threats, face higher risks of dying of the disease, of experiencing discrimination and a disproportionate impact as a result of confinement measures, and of being left without support to defend their peoples from intensifying rights violations even as the pandemic rages … COVID-19 has created an unprecedented wave of fear, sadness and hardship around the globe, yet indigenous peoples feel particularly forgotten and left behind (UN General Assembly, 2020: 5).

This invisibility is compounded by dispossession, displacement, assimilation, and cultural oppression for Indigenous peoples in urban areas in the United States and Canada. In both countries, settler colonial cities were formed historically on ancestral Indigenous peoples’ homeland, especially lands that afforded access to trade routes and other abundance. Other colonial policies and practices further compelled Indigenous peoples’ movements between rural and urban areas, but also the survivance with which Indigenous peoples have created contemporary urban communities (Howard and Lobo, 2013; Howard, 2019).

Special Rapporteur Calí Tzay asked for reports on COVID-19 related experiences of Indigenous peoples in the specific areas of access to health care, information, culturally appropriate services, justice, education, shelter, and sustenance. He also wanted to hear about Indigenous leadership in the implementation of national pandemic response, and the role of Indigenous healing models and traditional systems. We have taken up these areas of inquiry through the lens of Indigenous and human rights and a grounded methodology, referencing Indigenous community-based scholarship and comparative content analysis. The choice of documents and sources examined for our analysis in our original research was guided directly by the Special Rapporteur’s specific questions and included Indigenous and non-Indigenous COVID-19 information websites and policy statements issued by governments and organizations, media reports, review of Indigenous organization social media, webinars, and personal communications. Across these sources, we scanned for urban Indigenous content and the use of human and Indigenous rights discourse. In extensively expanding that work into the current article, we added to our analysis other written responses submitted to the Special Rapporteur’s call, which were selected for content from and about the United States and/or Canada with direct reference to urban Indigenous topics. In total eighty-four sources were examined in addition to the secondary literature cited.

## United States and Canadian Urban Indigenous Communities in Hemispheric Context

Indigenous peoples are interconnected through local, regional, and national experiences of the hemispheric dynamics of colonization and settler colonialism. Previous experiences of pandemics sickened and killed a large portion of Indigenous populations throughout the Americas since the arrival and colonization by Europeans. Today, movement and migrations over seasonal, short, and often extensive distances continue to be essential to Indigenous life throughout the hemisphere (Lobo, 2009). Despite continuing stereotypes that Indigenous peoples reside overwhelmingly in rural areas, since the early 19th century, they have moved from rural to urban areas within and across nation-state borders throughout the hemisphere, diversifying the Indigenous population that make up many cities.

The urban environment has not provided safe haven from COVID-19 for Indigenous peoples, and the impact has been devastating. In both the United States and Canada about three-quarters of all Indigenous people reside in urban areas (Rotondi et al., 2017; Congress of Aboriginal Peoples, 2019; Villarroel et al., 2020). As researchers who have focused much of our work on urban Indigenous topics for many years, we have observed and personally experienced the multiple effects, including impacts of and responses to COVID-19 for urban Indigenous peoples in the United States and Canada, while remaining cognizant of the hemispheric-wide domain within which the pandemic continues to unfold. There are varying degrees of health disparities experienced by Indigenous communities world-wide. However, as the World Health Organization’s (WHO) Commission on the Social Determinants of Health notes, the unhealthy life conditions endemic in many Indigenous communities is attributable to several chronic distal determinants, and “The inequity [in daily living conditions] is systematic, produced by social norms, policies, and practices that tolerate or actually promote unfair distribution of and access to power, wealth and other necessary social resources.” Colonization is one of the common underlying causes of persistent health disparities for Indigenous peoples (World Health Organization, 2008: 2).

This international guidance situates understanding of the impacts of COVID-19 for Indigenous peoples by highlighting specific reference to “the enjoyment of the highest attainable standard of physical and mental health without discrimination … the right of indigenous peoples to be actively involved in developing, determining, and administering health programmes through their own institutions … and to their traditional medicines and health practices” (UN General Assembly, 2020: 5). In taking up this framework, Special Rapporteur Francisco Calí Tzay introduced the central role of “development-related activities,” which include urbanization, in the displacement of Indigenous peoples across and away from traditional territories, and in the denial of their sources of nutrition and “symbiotic relationships with their lands, [which] has deleterious effect on their health” (UN General Assembly, 2020: 6). Moreover, the Special Rapporteur acknowledges that Indigenous “collective memory is marked by pandemics” and underscores the interconnection of systemic racism and inequities in health status, access to health care, and socioenvironmental factors that diminish immune response and expose Indigenous peoples more starkly to COVID-19 (UN General Assembly, 2020: 8; see also; Paradies, 2006a; Paradies, 2006b).

As the Special Rapporteur confirms, these are significant factors that exacerbate the way data is not collected and disaggregated in meaningful ways so that Indigenous peoples “continue to be invisible in the consciousness of majority populations and are likely to be left behind in prevention and care programmes and in the provision of other socioeconomic support” (UN General Assembly, 2020: 11). As Calí Tzay also states, “where communities distrust the Government or wish to assert exclusive ownership of such information [and where] indigenous communities have collected data themselves, [these] were not reflected or only partially reflected in national periodic COVID reports” (UN General Assembly, 2020: 11–12). This finding was noted as particularly problematic in urban areas where infection and mortality are not tied to Indigenous identification and therefore not only lead to invisibility in public records, but also expose “the lack of culturally specific approaches to health care in cities” (UN General Assembly, 2020: 12).

Overall, the Special Rapporteur’s reference to Indigenous peoples in cities is not expansive; however, his report asserts that poverty, overcrowded housing, and “deep racism and structural discrimination further hinders access to basic health and social services and protective equipment” in urban areas where one might assume these would be more available than in remote, rural regions (UN General Assembly, 2020: 10). Of particular concern are the escalation of violence against women and children in relation to lockdown policies, and other deleterious effects of restrictions on the freedom of movement of Indigenous peoples, with noted attention to those living in urban areas, and the increase of existing crizes in mental health and substance misuse (UN General Assembly, 2020: 8,17). The Special Rapporteur found that COVID-19 relief in the form of economic, health, and protective supports has been painfully protracted and insufficient given the predictability that Indigenous peoples would be disproportionately affected. While this probe found that in some cases funding was set aside for Indigenous peoples, it was routed in ways that compounded the exclusion of urban Indigenous communities (UN General Assembly, 2020: 14).

Moreover, responses largely excluded Indigenous peoples from leadership and coordination, and especially “failed to adequately take into account their specific needs across their various lifestyles or whether they live in … urban settings … [or] take the risk of contracting COVID-19 by traveling to cities on public transport to collect financial assistance to which they were entitled” (UN General Assembly, 2020: 14). The report emphasizes the particular economic hardships faced by Indigenous peoples in urban areas who rely heavily on informal and precarious employment conditions, have been disproportionately turned out of housing, are finding it difficult to access basic sustenance, and are subject to an intensification of stigmatization (UN General Assembly, 2020: 19). These conditions are additionally harmful in a context where the importance of kin and community-based supports, traditional extended family co-residence, and communal food sharing and the practice of spiritual ceremonies are often disrespected, while especially needed at this time of crisis (UN General Assembly, 2020: 8).

## United States Settler Colonial Policies Laid Bare in the Impact of COVID-19

In the United States, the known impact of COVID-19 on Indigenous populations overall is lacking although they are listed among the high-risk groups. The Kaiser Family Foundation estimates that American Indian/Alaska Native and Native Hawaiian/Pacific Islanders are at risk for contracting COVID-19 at a rate of 34% and 23% respectively, compared with 21% for Whites, 27% for Blacks, and 12% for Hispanics, between the ages of 18–64 (Artiga and Orgera, 2020: 3). Although the numbers lack completeness, disproportionate mortality rates are shocking in some areas such as New Mexico where Indigenous people account for 44% of COVID-19 deaths even though they constitute 11% of the state’s population, and Indigenous communities in Arizona have seen nearly 22% of COVID-19 deaths in that state where they make up 2% of the population. (National Congress of American Indians, 2020a: 2).

The poor health status of many Indigenous people is influenced by poverty, limited or lack of access to quality health care and the prevalence of chronic health problems or disabilities (Roubideaux and Dixon, 2001). In a nation with highly developed modern health care delivery systems, Indigenous peoples often face numerous barriers to disease prevention and quality health care, including lack of health insurance, structural racism, mistrust, and other factors such as access to technology. The convenience of working remotely is not possible for many Indigenous peoples employed in the service industry and/or other labor occupations (U.S. Census, 2010). They comprise a high percentage of front-line workers whose employment poses unprotected exposure to COVID-19. In some areas of the country, these workers commute daily between rural and urban communities to work, to shop, to attend school or attend recreational activities; and in doing so, they are at risk for developing or transmitting the virus. For example, when the first case of COVID-19 was confirmed in the Navajo Nation, it was traced to an earlier evangelical church gathering that included reservation and off-reservation attendees (Lee, 2020).

While Indigenous people make up only about 1.7% of the United States population, some estimate that as many as 80% live in urban or off-reservation communities (Villarroel et al., 2020). For some younger generation, urban life has and continues to be their only living experiences. Despite their places of residence, tribal members who have federal recognition have certain rights that are retained and secured through treaties with the United States. The migration to cities for many Indigenous families has occurred over time. For example, some individuals or couples were relocated to the city from reservations under a federal relocation program in the 1950s and 60s (American Indian Policy Review Commission, 1976). The relocation program recruited young adults with promises of employment or job-training opportunities in selected cities. However, these promises were not realized, and instead, resulted in Indigenous people living in segregated neighborhoods, being assigned to poor or substandard vocational training that resulted in joblessness, poverty, and sometimes poor health (Lobo and Peters, 2001). Other Indigenous people elected to live in cities after military service or as graduates of forced off-reservation boarding school experiences (Howard and Lobo, 2013).

Seeking a place of acceptance in an unfamiliar urban environment, organizations in some cities established a safe gathering place such as the Oakland Intertribal Friendship House in California, or the Chicago American Indian Center in Chicago, IL (Online Archives of California, American Indian Community History, 2021). These organizations focused on creating protected places within the often threatening and racist urban environment; places to gather, form friendships, create social alliances, eat together, locate and share information and resources including health care, food and housing, economic opportunities, education, and to practice cultural life. These resources became powerful forces in creating and sustaining Indigenous urban communities and advocating for the assertion of Indigenous rights for Indigenous peoples living in cities. Local groups formed, like for example, the Chicago American Indian Community Coalition, and national coalitions grew such as the National Urban Indian Family Coalition that includes twenty-four cities and forty-five urban Indigenous organizations (National Urban Indian Family Coalition, 2020: 8; Online Archives of California, American Indian Community History, 2021).

### The Historical and Disease Context for COVID-19

Mistrust reflects the devastating history left by cycles of epidemics that followed European contact. Lacking immunity and weapons for protection, survivors faced dispossession of their ancestral homeland and most were forced into resettlement onto small plots of land (federal reservations), with little or no resources for sustainability. Warfare and epidemics are remembered as instruments of genocide that hastened the depopulation of the Americas as well as the decimation of many Indigenous groups. This includes threats of biological warfare. For instance, a proposal by an officer during the conflict over American land between England and France to distribute smallpox contaminated blankets to tribal warriors remains widely remembered among Indigenous people today (Jones, 2004: 94–95). Mistrust has also been steadily reinforced by policies of land dispossession and forced removal that ignited and expanded the erosion of ancestral Indigenous cultural history and strength (Lindquist and Zanger, 1993). The removal of Indigenous peoples to the most unproductive land and where they remained contained became ideal breeding ground for more epidemics, poverty, malnutrition, and ill health.

Tribes negotiated treaties with the federal government to secure Indigenous rights, as well as various forms of payment, including health care for ceding land. Treaty terms have rarely been honored by the government. In fact, history has often reflected lack of good faith, leadership and guidance in the way federal authorities have failed in their treaty responsibilities. Initially, the United States established a small Indian agency within the War Department, a federal agency responsible for warfare and containment, not health care. When the United States Department of Interior was established in 1849, the health care responsibility for Indigenous peoples was transferred to this new agency, but with no additional resources. Annual federal appropriations to support the programs in the Indian Affairs office in the Department of Interior were not only inadequate, but each dollar was earmarked for specific items, many of which were never purchased (Joe, 2003).

Over time, the health conditions of Indigenous peoples continued to decline while no substantial resources were implemented (Meriam, 1928; U.S. Commission on Civil Rights, 1966; American Indian Policy Review, 1977). Instead, the government in the 1950s once again moved the health responsibility for Indigenous peoples from the Department of Interior to the United States Public Health Service (PHS). Although there have been some improvements, a series of civil rights reports, some commissioned by the government, have repeatedly confirmed that the poor health conditions of Indigenous peoples had not greatly improved due to the lack of funding and meaningful policies or legislations (U.S. Commission of Civil Rights, 2003; U.S. Commission of Civil Rights, 2018). In 2017, Indian Health Service (IHS) expenditures per patient was reported to be $2,834 compared to $9,990 per person in federal health care spending nationwide (U.S. Commission of Civil Rights, 2018: 66). In addition, the funding allocated for urban Indian health programs is estimated to be only 22% of projected need (U.S. Commission of Civil Rights, 2018: 74). The urban health programs’ share of IHS funding has not reflected the demographic shift from rural to urban communities (Artiga and Arguello, 2013).

Urban or rural, Indigenous populations in United States are haunted by multiple health disparities (Joe, 2015). The leading causes of death for Indigenous people include heart disease, malignant neoplasm, unintentional injuries, and diabetes (Indian Health Service, 2019). Until the mid-1950s, infectious diseases such as *tuberculosis*, glaucoma, and meningitis were the leading causes of death and hospitalization. Although these have declined, non-communicable diseases with an array of complications such as high rates of chronic liver disease and chronic lower respiratory diseases have escalated (Indian Health Service, 2019). Some infectious diseases are still prevalent as well as new ones including antibiotic resistant *tuberculosis*, HIV/AIDS, invasive pneumococcal infections, and diarrheal infections (Butler et al., 2001; Holman et al., 2011; Cheek et al., 2014).

### Accessing Health Care

The rights of access to health care for Indigenous populations living on federal tribal reservations is determined by an array of local, state, or federal eligibility criteria, some imposed to limit as much as possible the exercise of these rights. For example, to be eligible to receive health care provided by the IHS, an individual needs to be enrolled in a federally recognized tribe (National Congress of American Indians, 2019: 11). Not all tribes have federal recognition; some have only state recognition and are not eligible for health care provided by IHS (James et al., 2009). Medicare and Medicaid provide other primary health care coverage for both reservation and urban-based Indigenous populations. IHS has its own longstanding medical data system in place and draws on this database to mark progress and document unmet health care needs for reservation-based populations only. A similar comprehensive system does not exist for Indigenous populations in urban communities.

This gap in services has led to the development of urban Indigenous health care organizations. Many followed the pattern of free store front clinics in the 1960s, which operated part-time and depended on donations and volunteer providers. The first clinics were established in San Francisco, CA, Seattle, WA, Minneapolis, MN, and Oklahoma City, OK. The services provided at these sites were either free or offered on a sliding scale. Because IHS could only provide health care to reservation-based tribal members, no federal assistance was available to these urban clinics until Title V of the Indian Health Care Improvement Act (P.L. 94–437) was passed by Congress in 1976. Even so, the Act authorized approximately 1% of IHS funding for urban clinics, which has not increased to meet the health care needs of the 80% of Indigenous peoples living in cities. The annual congressional appropriation for IHS has been and continues to be discretionary and is not an entitlement as it is for other federal programs such as Medicare. Inadequate funding prevented most storefront clinics from providing a full range of medical care until the scope of work for some clinics was expanded in 1988 with an amendment to the Indian Health Care Amendment (P.L. 100–713). The passage of the 1988 Anti-Drug Abuse Act also gave urban health programs the ability to deliver alcoholism and substance-misuse treatment and prevention programs (Namis, 2019).

### Response to COVID-19

The first reported cases of COVID-19 in the United States were in Seattle, where the local Indigenous urban health programs serve about 6,000 patients annually (Ortiz, 2020). Following on the health rights history and context just described, these programs were not equipped nor had the resources to assist their patient population as the pandemic hit, a pattern repeated across North America (Crevier, 2020). Lack of funds and medical equipment disrupted or ceased some essential services provided by urban Indigenous health programs. The chaotic situation everywhere meant few supplies or equipment were available to small, non-profit clinics or health programs. In one case, an Indigenous clinic in Seattle requested medical supplies in March 2020, only to get a box of body bags the following month. The shipment was said to be unintentional, but when it arrived, the clinic’s Chief Medical Officer said: “My team turned ghost white, we asked for tests and they sent us a box of body bags” (Ortiz, 2020).

The virus-related closing of other key urban resources was devastating for urban families, especially those who depended on them for many services. On average, the forty-five urban Indian organizations that are members of the National Council of Urban Indian Health serve approximately 77,000 patients annually. Reimbursement funds for their services are received from state and local governments, but the forced closure of these services threatened their survival (National Council of Urban Indian Health, 2020). For example, one program in St. Paul, MN, reported that their emergency shelter was losing $14,000 a month due to stay-at-home and physical distancing requirements (National Urban Indian Family Coalition, 2020: 6). As the volume of patient load plummeted, the reduced services meant many of the health programs could not apply for third party insurance payments, a resource for over half of the budgets for many programs (National Council of Urban Indian Health, 2020). Kerry Hawk Lessard, Executive Director of Native American LifeLines, serving Baltimore, MD and Boston, MA, reported that because the program does not deliver direct medical services it could not offer COVID-19 testing. However, the program staff did not abandon their responsibilities, and called all their 770 patients to “ensure they know about COVID-19 risk, symptoms, and prevention and to understand the rates of positive cases and mortality….” (Hlavinka, 2020a).

As the pandemic wore on with social distancing, quarantines, and the temporary closing of many urban Indigenous organizations, use of the internet to reach community members and for inter-agency communication expanded significantly. Essential information regarding resources, events, personal announcements and so forth that has been a rich part of in-person urban Indigenous organization experience increasingly took place through social media and online presence. For example, in Phoenix, AZ, the Indigenous health organization newsletter *Native Health News* had 57 internet pages in its September edition, and included extensive COVID-19 health tips and information regarding regional health and medical resources, tele-counseling, schedules for home food delivery, safe children's activities and programs (Native Health Phoenix, 2020).

Urban Indigenous communities were devastated by the sudden closing of their health and social service organizations. The Tucson Indian Center, in Tucson, AZ, locked its doors and posted emergency phone numbers, while shifting to serving clients remotely. Community events or celebrations were canceled but staff quickly reorganized to deliver food, medicine, and other necessities to those most in need. A major national Indigenous newspaper*, Indian Country Today*, started listing many important events or Indigenous enterprises that had been closed, canceled, or postponed, including special sacred ceremonial activities. By early June, some organizations were carefully beginning to return to limited activities, but the closing and cancelations impacted basic rights to practice cultural activities, spiritual well-being, and economic sustainability for urban Indigenous communities (Shah et al., 2020).

In Chicago, where the Indigenous population is approximately 27,000, researcher Larkin-Gilmore commented on how blame for COVID-19 is placed on “native bodies [as] somewhat more susceptible to COVID-19 when really the reasons for that is far more complicated and based in … structural and political disparities” firmly rooted in racism (quoted in Spoto, 2020). Moreover, because Illinois counts Indigenous people as “others,” the true picture of COVID-19 mortality and morbidity is lost in the data. The director of the urban clinic in Chicago reports that between the end of April and June 2020, 23% of the clinic’s Indigenous patients tested positive for the virus, a rate that is 7.5% higher than for other Chicago residents. Data obtained through the Freedom of Information Act, for a period between March and April 2020, showed a 15% coronavirus mortality rate for Indigenous people compared to 5% for the general population of Chicago (Spoto, 2020). These examples illustrate how deeply rooted erasure of basic human and Indigenous rights fatally manifest on Indigenous bodies in urban areas.

### Indigenous Population Statistics, Health and Data Issues

Once registered in the mainstream health care delivery system, Indigenous patients’ ethnicity is often misclassified. It is not uncommon for a death certificate, or other racial or ethnic classification data entered on health records, to identify an Indigenous person as “white or unknown” (Centers for Disease Control, 1993; Haozous et al., 2014). The increase in multi-racial admixture for Indigenous peoples, especially those living in urban areas, adds to the complexity of racial/ethnic identity. Misclassification treats Indigenous people as an “invisible population” (Lobo, 2002; Liebler, 2018), and reflects persistent population debates where even the pre-contact population of Indigenous peoples and the extent of depopulations associated with epidemics and warfare continue to be questioned, although careful scholarship has estimated genocidal impacts on Indigenous demographics (Dobyns, 1983; Thornton, 2000; Jones, 2004).

Several local and national policies have mandated health surveillance systems with specific requirements on data collection, reporting, and data sharing. Included are required reporting of deaths, births, hospitalization, and other reportable data used to track and evaluate changes in the health status of the population. While data reported by surveillance systems are reliable and useful, these can also pose problems when some important health data is not available (Jim et al., 2014). With COVID-19, the NCAI reported 42,085 Indigenous people diagnosed between March 23 and September 1, 2020, relying on IHS data (National Congress of American Indians, 2020b). The NCAI also indicates that the highest COVID-19 incidence rates among the Indigenous populations were in the Navajo Nation and two urban communities—Phoenix, and Oklahoma City. While this and other IHS data are important, data for Indigenous people living off-reservation or in urban communities are not routinely included. The establishment of 12 IHS-funded regional Epidemiology Centers is slowly changing and improving Indigenous health data, with one center focused on urban Indigenous health programs. In 2000, the Seattle Indian Health Board, home of the Urban Indian Health Institute (UIHI) was funded to establish this center, and it serves or collaborates with 62 urban-based programs nationwide. The Center’s goal is “to decolonize data for indigenous people, by indigenous people.” Although all the Epidemiology Centers have been granted official Public Health Authority by CDC, during the pandemic most national and state agencies refused to share or provide COVID-19 data with these centers; the typical reason for refusing was: “data is nonpublic” (Tahir and Cancryn, 2020).

For several decades, the problem of racial misclassification of Indigenous people, especially in cities, has hidden the health problems confronting Indigenous populations. Several states label American Indians as “other” including Texas, Florida, Michigan, New York, and California. Thus, for cities with large Indigenous communities like New York, NY, and Los Angeles, CA, also heavily impacted by COVID-19, there is no explicit data breakdown about Indigenous populations (Nagle, 2020). This leads to misinformation and diminishes understanding of the extent and significance of the impacts of COVID-19 for Indigenous peoples in urban areas. The missing data is a critical concern for Indigenous agencies working to address health disparities. This problem is not ignored by data keepers. For one, the CDC's Morbidity and Mortality Weekly Report (MMWR) found COVID-19 cases for United States’ Indigenous population was 3.5 times higher than for non-Hispanic whites (Hatcher et al., 2020). The report was based on data obtained from only 23 states that routinely record race and ethnicity. This excessive absence of data represents an important gap in public health data and additional resources to support case investigation and reporting infrastructure in Indigenous communities are needed (Hlavinka, 2020b).

Even with these data problems, the United States Centers for Medicare and Medicaid Services (CMMS) reported in July 2020, that Indigenous people had the second highest rate of hospitalization for COVID-19 among racial/ethnic groups after Black Americans. One CDC report found that from among 340,059 confirmed COVID-19 cases, the rate of infection was 3.5 times higher for Indigenous people than it was for the population identified as white. This report also indicated infections were more common among younger Indigenous people, with a median age of 40, compared to 51 years for whites (Smith-Schoenwalden, 2020). Indigenous patients may also require complicated treatment for COVID-19 due to high co-morbidity rates. Hospitalization for COVID-19 care is extremely expensive. The CMMS paid $2.8 billion in Medicare fee-for-service claims for patients hospitalized with COVID-19 at that reporting time, with the average payout at $25,255 per beneficiary (Centers for Medicare and Medicaid Services, 2020).

One Indigenous physician providing care at a Navajo Nation hospital notes that the pandemic further exposed all the deficiencies and inadequacies that were accepted as ‘normal’ pre-COVID-19 such as poor access to water, electricity and transportation (Krisst, 2020). Many of the historical, structural, and contemporary inequities of settler colonial society are manifest in the inadequacies of the health care delivery systems serving Indigenous peoples in the United States. Yet, even as COVID-19 makes more starkly visible the reach of these inequities into the growing social, cultural, and economic divide of suffering experienced by Indigenous peoples, urban Indigenous peoples remain stubbornly invisible.

## Pandemic Colonialism in Canada

This “normalization” of the structural oppression and invisibility of urban Indigenous peoples just described for the United States is mirrored in Canada even if the events that trace the patterns of settler colonialism and articulation of Indigenous rights are slightly different. Sixty-five to eighty percent of the two million Indigenous people in Canada live in urban and “off-reserve” communities (Rotondi et al., 2017; Congress of Aboriginal Peoples, 2019). Information on COVID-19 for Indigenous people living in these areas is not gathered effectively by the Canadian state. This is complicated by the overall problem that “no agency or organization in Canada [is] reliably recording and releasing COVID-19 data indicating whether or not a person is Indigenous” (Skye, 2020). Indigenous Services Canada (ISC) officially collects data for First Nations communities that explicitly excludes urban communities and individuals living off-reserve and in the territories. ISC relies on each “provincial chief public health officers work with ISC's regional medical officers and nurses to provide medical support as needed when a positive case is reported” (Indigenous Services Canada, 2020). The Public Health Agency of Canada (PHAC) reports daily on COVID-19 rates for the country but includes disaggregated data on age and sex only, referring visitors to links to provincial and territorial agencies for more details. (Public Health Agency of Canada, 2021). These, in turn, vary in their reporting on Indigenous peoples’ health status, and generally do not publish data for Indigenous cases.

ISC may track Indigenous individuals diagnosed with COVID-19 who are members of First Nations and registered with the Inuit Health Board living in urban areas, but these are not distinguished as such. ISC has admitted that data collection needs to be improved on COVID-19 and is investing in alternative approaches. However, the ISC plan focuses on the idea that it is the remoteness and isolation of Indigenous peoples in Canada that is the barrier to accurate data collection, with little attention to urban populations in these efforts, even as urban Indigenous representative organizations are ready and available to engage (National Association of Friendship Centers, 2020). While Canada claims it is acting on COVID-19 through engagement with Indigenous leadership, respect for treaty rights, and ending enduring inequities grounded in structurally racist policies, funding in practice is focused on immediate pandemic relief and mitigating risk (UN General Assembly, 2020: 12; Government of Canada, 2020). The state thus exercises pandemic colonialism, sustaining settler colonial status quo through a method of pairing intervention on proximal aspects of the pandemic with talk about structural oppression and Indigenous rights.

State relationships to Indigenous peoples are meant to be guided by the historically and constitutionally grounded fiduciary duties of the federal government to recognize and support Indigenous peoples’ self-government. However, this duty is diluted by the extent to which federal responsibility has devolved over decades to provincial, territorial, and local entities which handle health care monitoring and delivery. This has entrenched settler colonial erasure of Indigenous peoples’ health status. The census and other data collection mechanisms have been criticized for a long time with regards to inaccurate counting of urban Indigenous peoples (Howard and Lobo, 2013; Rotondi et al., 2017). It is a systemic problem that extends from faulty data collection even for Indigenous peoples who are counted, highlighted by the COVID-19 crisis (Dunne, 2020a; Dunne, 2020b). For example, the Aboriginal Peoples’ Survey (APS), conducted every five years focuses on education, employment, health, language, income, housing and mobility, but does not reach urban communities sufficiently, not counting people experiencing homelessness or living in non-permanent or collective dwellings. Statistics Canada promises that its alternative crowd-sourcing survey collection series includes information that will provide better understanding of these “hard-to-reach” populations, and how COVID-19 is experienced by urban Indigenous communities. To date these data remain insufficient or are hard to extrapolate. The scattered and insufficient ways data is collected about Indigenous peoples by the state is correlated to funding that does not meet the needs of urban Indigenous communities and constitutes a form of discrimination deeply ingrained in differential rights of acknowledgment as detailed below.

### Disseminating Information to Urban Indigenous Communities in Canada

The PHAC funds the National Collaborating Center for Indigenous Health that provides a clearing house of COVID-19 resources ranging from general guidelines to podcasts, webinars, and fact sheets aimed at Indigenous individuals and communities though none are specifically urban. ISC did create preventative public service announcements about COVID-19 for radio available in 20 Indigenous languages. Each province’s health authority maintains websites with information specific for Indigenous peoples with content from general guidance on avoiding disease spread to detailed Indigenous-engaged advice specific to Indigenous concerns, some in Indigenous languages. While we did not examine the impact of providing COVID-19 information in Indigenous languages, the value and significance of doing so is reported from a global standpoint at the website of the UN International Year of Indigenous Languages (IYIL n. d.) and merits further study.

Urban Indigenous communities are not differentiated at the government websites examined, however, some of the advice such as from Indigenous physicians on ceremonies, for countering stigma, and keeping Elders safe, would be useful in urban contexts if Indigenous peoples can find them. Some of the Indigenous provincial and territorial-level health authority counterparts include attention to urban Indigenous communities. For example, the First Nations Health and Social Secretariat of Manitoba maintains a COVID-19 Pandemic Response Coordination Team and provides detailed reporting on cases including for the province’s major city of Winnipeg which has the largest urban Indigenous population in Canada. The Métis Nation of Canada (MNC) is a “distinct Indigenous people and nation recognized in the Constitution Act 1982,” with many constituents in urban areas. The MNC issues daily COVID-19 messages and provides an extensive clearinghouse of information at its website. However, these may not be accessed by other Indigenous peoples.

### Urban Indigenous Input on National Response and Programs that Affect Them

At the end of May 2020, the federal government pledged 90 million dollars to support off-reserve Indigenous organizations, increased from 15 million announced in March. The National Association of Friendship Centers (NAFC), which represents over 100 centers serving urban Indigenous people across Canada, spoke out about how urban Indigenous people were being left behind by the Canadian federal government’s response to COVID-19, and that the funding promised did not cover all the needs across Canada. Even with the increased funding, the amount designated for urban and off-reserve communities is less than 10% of the overall amount of COVID-19 support allotted for Indigenous peoples. The NAFC emphasized how this highlighted the Canadian government’s lack of acknowledgment of urban Indigenous peoples, and its disregard for their constitutionally and internationally protected rights (Canada’s National Observer, 2020).

The NAFC also submitted a response to the UNHRHC Special Rapporteur, which noted that while the NAFC “continually provided context from the urban perspective and realities” the impact is variable and there remains a “large gap in urban Indigenous voices and perspectives being heard” (National Association of Friendship Centers, 2020). The NAFC submission goes on to emphasize the danger posed to accessing potentially life-saving services caused by the entrenched “jurisdictional wrangling,” noted above. This frustrating business-as-usual approach by the state to the relationship with urban Indigenous leadership means that securing funds for urban Indigenous communities is not only delayed, it is “not based on need or population” (National Association of Friendship Centers, 2020: 3). The fact that federally recognized Indigenous governments receive funding directly based on population, whereas urban Indigenous representative organizations have to competitively apply for grants lays bare the discriminatory nature of the state’s policies and practices toward Indigenous peoples in urban areas. The NAFC called this “flawed and disrespectful” and underscores how the Canadian false dichotomy that distinguishes rural and urban Indigenous populations hurts Indigenous peoples in totality. The United Nations may agree, writing in a 2010 report about how this distinction globally serves the assimilationist interests of nation-state building (United Nations, 2010). It may be further argued that this approach violates the international conventions mentioned above, and to which Canada is a signatory, including the United Nations Declaration on the Rights of Indigenous Peoples, officially adopted by Canada in 2016.

The Congress of Aboriginal Peoples (CAP) which represents “the interests of Métis, non-status Indians … and all off-reserve status and non-status Indians” filed a legal action “over inadequate and discriminatory funding during COVID-19” in mid-May 2020 (Congress of Aboriginal Peoples, 2020). The court application framed the problem in terms of discrimination and violation of Indigenous rights enshrined in the Canadian Charter of Rights and Freedoms, and in Supreme Court precedent. In a press release, CAP wrote that “despite the federal government’s laudable goals, the funding allocations have been discriminatory and at the expense of the doubly disadvantaged Indigenous populations served by CAP.” The release further remarked on the Canadian government’s disregard of CAP efforts to be heard and specified the urgency with which support is needed for health care including mental health, but also to address housing, education, food insecurity, and the special needs of Elders (Congress of Aboriginal Peoples, 2020).

In addition to these urban-specific organizations, the Assembly of First Nations, (2020), a national advocacy organization for First Nations’ citizens, included urban Indigenous people in one of its several COVID-19 podcasts. However, as the national representative organization for reserve-based communities, the AFN has not generally used its power to tackle the rural-urban divide centrally taken up by the NAFC and CAP. Overall, the federal government funds acknowledge two major issues in Indigenous communities that have been exacerbated by COVID-19: the need for long-term income-support and to build and support shelters for women fleeing escalated domestic violence. This followed on news that Indigenous women’s shelters closed, including one in Montreal, after “multiple attempts to get local health and social services personnel to come test residents, and a frustrating ordeal getting assistance” (Rowe, 2020). Extraordinarily, however, the NAFC reports that all of the shelter funding announced excludes urban Indigenous communities, despite the fact that “Indigenous women and their children face violence and need shelter in off-reserve contexts, and irrespective of whether they are First Nations, Métis, or Inuit” (National Association of Friendship Centers, 2020: 7).

### Pandemic Exacerbation of Intersectional Discrimination and Human Rights Infringements

The above matters of discrimination that remain pervasive in Canadian policy and practice based on the on-reserve/off reserve distinction also ignores a deeper intersectional analysis that takes into account the multiple social identities that shape Indigeneity and the mutually reinforcing inequities that co-constitute numerous systems of oppression. Indigenous peoples’ engagement with urban places and urbanization processes do not stand separate from socio-economic structures, gender and age diversity, and disability status, among other intersections. This exacerbates the impacts of COVID-19 if no relief is aimed at the particular systemic barriers faced by youth, Elders, and disabled, or for “Two-Spirit and LGBTQ + individuals, who are at higher risk for infection due to rates of poverty, social isolation, and systemic discrimination within the health care system…. Including an intersectional lens to distinctions will better position the Government of Canada to provide urban indigenous community members with the supports they need throughout and beyond this pandemic” (National Association of Friendship Centers, 2020: 5).

The PHAC identifies a series of “significant economic, social and health challenges that could play a role in how COVID-19 affects the lives of Indigenous people” including in urban settings. These are high levels of chronic disease, inadequate housing, multi-generational cohabitation, as well as isolation from, and discrimination in, health care facilities. The federal government also commissioned a brief study that correlates socio-economic data collected in the 2016 federal census and 2017 APS to known potential risks and challenges of COVID-19. This study emphasizes that high rates of poverty, food insecurity, and the inability to cover an unexpected expense make Indigenous people, especially women and children in urban areas very vulnerable to immediate and long-term impacts of COVID-19, including slowing the spread of disease, stunting access to education, and increasing the risk of homelessness (Arriagada et al., 2020). In its submission to the UNHRHC Special Rapporteur, the Government of Canada confirmed these particular differential impacts for what they called “intersectional and unique sub-populations” (Government of Canada, 2020: 8). The report shares plans to initiate “Indigenous-led engagement related to a continuum of supportive care services … to further inform the comprehensive community-based approaches that support the most vulnerable Indigenous people,” however, it generally steers clear of any detailed discussion of urban Indigenous community concerns, focusing on the direct effects of isolation and shutdowns of services and businesses (Government of Canada, 2020: 8).

Meanwhile, it appears that some provinces to whom the federal government devolves responsibilities for many of these issues, identify more directly the structural contexts for COVID-19’s differential impacts on Indigenous peoples. As part of a global analysis of social determinants and COVID-19, Public Health Ontario acknowledges that Indigenous “communities face health inequities associated with complex influences of colonization, residential schools and continued experiences of systemic racism” and references other research on these issues that includes urban sources (Reading and Wein, 2009; Beckett et al., 2018). While this provincial recognition is significant it may validate federal reneging on its Indigenous treaty-based responsibilities and translate into inappropriate or insufficient support.

The Native Women’s Association of Canada (NWAC) also submitted a response to the UNHRHC Special Rapporteur, which provides results of its survey (n = 750), “The Impacts of COVID-19 on Indigenous Women and Gender-Diverse People in Canada.” The submission centers on NWAC’s ongoing work to hold the Canadian government accountable to its national action plan to attend to the “Calls for Justice” issued in findings of the National Inquiry on Missing and Murdered Indigenous Women (National Inquiry into Missing and Murdered Indigenous Women and Girls, 2019; Native Women’s Association of Canada, 2020a). The survey illuminates the glaring correlation between state responsibility for the ever-expanding violation of Indigenous people’s “absolute right to physical and mental integrity,” and a clear upsurge in domestic violence in conjunction with the conditions of COVID-19. The survey concludes, “actions from government at all levels are needed to address the issue of systemic violence against Indigenous women and gender-diverse people.” Again, in its submission to the UNHRHC, the Government of Canada appears more focused on a proximal understanding of violence particular to the temporary context of the pandemic. Referencing its “engagement strategy” and national action plan, it states that the “challenges of family violence and mental health during isolation, or the needs of LGBTQ and Two-Spirit individuals or those with disabilities requires particular attention during the pandemic” (Government of Canada, 2020: 8).

As a national advocacy organization and clearinghouse of information, the NWAC provides an extensive list of resources in the context of messages from the association’s president and in-house Elder who acknowledge the particular struggles of women and Two-Spirit people, including in urban areas. They state, “disease outbreaks affect women and men differently, and women and gender-diverse people are far worse off, especially when it comes to treatment and care.” (Native Women’s Association of Canada, 2020b). They also draw attention to the way women’s roles as caretakers of people and in relationship with water, which has been in a contamination crisis also largely ignored by the federal government, complicates their positions in relation to the pandemic (see Human Rights Watch, 2020; Thompson et al., 2020).

The National Aboriginal Council of Midwives (National Aboriginal Council of Midwives, 2020) has developed considerable information on how COVID-19 not only compromises pregnancy and birth, but also the care midwives provide “beyond the clinic” including for Elders and others who are immune-compromized, “living in overcrowded homes, struggling with mental illness, substance use and trauma.” Their work has increased awareness “of the inequitable burden of COVID-19 on marginalized populations” including amplification of “already inequitable access to safe sexual and reproductive care … [and] how racism contributes to ill health and lack of access to healthcare” (Toronto Foundation, 2020). Discrimination and racism against Indigenous peoples in urban areas has been already widely reported and exposed in health care (Allan and Smylie, 2015; Brian Sinclair Working Group, 2017). The alarm has been raised by Indigenous leaders about how fears and lack of trust present barriers to Indigenous people using mainstream health care services, which can translate into waiting with COVID-19 symptoms, avoiding testing and potentially vaccination. It is more urgent than ever that Indigenous organizations are supported in administering testing and health care services themselves, as well as being fully engaged in critical care settings and vaccination delivery (Waakebiness-Bryce Institute for Indigenous Health, 2020a; Waakebiness-Bryce Institute for Indigenous Health, 2020b).

### Urban Indigenous Initiatives and Traditional Practices

To best understand the impacts of COVID-19 for Indigenous peoples in urban areas, look to the initiatives of Indigenous social movement organizations which have worked since the mid-20th century to deliver culturally appropriate services to this sector of the population and which form the basis of vibrant, self-determined urban Indigenous communities. (Howard and Proulx, 2011; Howard and Lobo, 2013). There are many concerns clustered at the top of the lists of urban Indigenous community organizations about COVID-19: homelessness or instable housing, crowded housing, opioid use, food insecurity, mental health issues, trauma, the inability to access cultural, Indigenous-led services, and higher rates of chronic disease associated with COVID-19, severe complications and death.

Urban Indigenous organizations have repeatedly noted that they faced difficulties finding help when the outbreak started, and devised plans to care for the community on their own (Waakebiness-Bryce Institute for Indigenous Health, 2020a; Waakebiness-Bryce Institute for Indigenous Health, 2020b). While this translated into insufficient and late-coming funding noted above, the crisis has illustrated how vital it is for Indigenous people to lead, and for those controlling non-Indigenous resources to support Indigenous community leadership. For example, NACM emphasizes the long-standing colonial inequitable system in which midwives operate, their crucial role as advocates for reproductive justice and as carriers of deep ancestral knowledge, key to mediating spiritual and mental well-being amid the pandemic. In the urban context, Seventh Generation Midwives in Toronto illustrate how important these dimensions of their work are in their adaptations to the pandemic, emphasizing their approach to “care through the lens of social justice … ” Also in Ontario, the Alliance for Healthier Communities includes ten Indigenous community-led primary health care organizations that “provide a combination of traditional healing, primary care, cultural programs, health promotion programs, community development initiatives, and social support services.” One of these, Anishnawbe Health Toronto, started a “Mobile Healing” RV to provide COVID-19 testing as well as its other services including primary care.

Na-Me-Res, a shelter for Indigenous men in Toronto, is one of over 40 Indigenous organizations in that city. The shelter already had a pandemic plan in place when two cases of COVID-19 occurred in late March. They implemented action even before the WHO declared the pandemic. Na-Me-Res shared this plan within the network of shelters throughout the province and coordinated with other Indigenous organizations in the city through long-standing relationships of cooperation. However, Na-Me-Res also reported that useful help was not forthcoming from mainstream public health agencies which advised isolation, impossible to implement in the 71-bed shelter. The management team quickly organized to act without outside help and successfully remained free from further COVID-19 infection. “We are basically alone in this,” executive director, Steve Teekens noted in May 2020. He also found assurance in the strengths exhibited by the initial community-based response, “We have been through many pandemics in our history here on Turtle island and I believe we will get through this one too” (Waakebiness-Bryce Institute for Indigenous Health, 2020b).

Organizations specifically serving Indigenous LGBTQ people also offer considerable insights into effective holistic prevention and care that builds on historic experience with HIV, and anti-stigma/anti-discrimination activism. For example, the organization, 2-Spirited People of the first Nations, located in Toronto, first posted community-specific information to Facebook on March 9 and continued to safely carry out their food help and harm reduction programs after their offices closed. They and many other urban Indigenous community organizations rapidly adapted programming to social media and YouTube to include new online integrated emotional, mental, spiritual, and physical health services, and links to supports such as the Two Spirit Journal’s (2020) series, “In Our Own Voices: Two-Spirit People Responding to Covid-19.”

Indigenous health research organizations are responding to the immediate need for information as well as considering long-term impacts and strategies emergent from the COVID-19 crisis. The Waakebiness-Bryce Institute for Indigenous Health and Well Living House use Facebook Live, and a YouTube channel to post basic information, as well as to feature speakers from the frontlines of Indigenous organizations to convey their needs and long-range perspectives. Highly important in-person cultural services and programming were canceled and postponed, but many urban Indigenous organizations adapted quickly, extending cultural services through telehealth technologies, and tools like Facebook Live and Zoom. Online prayer services, drum socials, craft classes, and other forms of engagement keep cultural contact going to curb the negative impacts of social distancing. Staff at the Native Women’s Resource Center (NWRC) in Toronto shifted to the phone and online quickly to ensure that the women and children they serve did not experience a discontinuation in the sense of community they usually enjoy within the walls of the center. This included access to healers, spiritual, and cultural service providers through online group and individual appointments. NWRC also considers care for staff wellness, taking actions like supplying medicines and teas to use as they work from their homes. Weekly ceremonies such as sacred fires have continued in the city with creative suggestions like lighting a candle at home at the same time as these fires are started to maintain that connection (Waakebiness-Bryce Institute for Indigenous Health, 2020b). Even as these efforts may not reach the most vulnerable for whom the precarity of isolation, insecure housing, and exacerbated mental health and well-being, prevent access, they highlight the importance of Indigenous leadership and culture in COVID-19 short and long-term response.

## Conclusions and Actionable Recommendations

This research originated with a briefing paper submitted in response to the interest of the United Nations Human Rights High Commission Special Rapporteur on the rights of Indigenous peoples, Francisco Calí Tzay, to understand the impacts of COVID-19 in Indigenous communities. Our expansion on that document here exposes the extensive shortcomings associated with existing policies and practices which have accelerated COVID-19 morbidity and mortality for urban-based Indigenous people in the United States and Canada. We have situated these shortcomings the contexts of historical and ongoing settler colonialism, intersectional discrimination and human rights infringements, and the strengths of Indigenous urban community-building. We have underscored the longstanding lack of national uniform policies, rooted in the violation of Indigenous peoples’ shared, historical nation-to-nation treaty-based and constitutionally protected rights and relationships as these extend to urban areas. The pandemic reveals how the United States and Canada, both settler-colonial nation-states, have each shirked responsibilities enshrined in these relationships, infringing on specific Indigenous rights, and more broadly defined basic human rights. These responsibilities extend not only to addressing biomedically defined health needs but, to overall community well-being through concrete actions against racist and misogynistic structures that perpetuate intersectional discrimination and human rights infringements. These include structures that sustain and escalate racial and gender-based violence, cultural disenfranchisement, and the dispossession of Indigenous peoples from the life-giving nourishment of their relationships to their lands. These basic nation-state responsibilities cannot be applied or neglected based on a distinction between the rural or urban location of Indigenous peoples.

The UNHRHC Special Rapporteur reminds states of this obligation under the authority of the international covenants to which they are signatories, when including among its seventeen recommendations, that states provide timely support and funding for prevention and broad care services identified by urban Indigenous communities and carried out through self-determined initiatives. Urban Indigenous leadership in both the United States and Canada have clearly stated the means through which to make this central recommendation actionable. Our research brings to light specific actionable recommendations in three key areas of public health, funding, and culture.

First, public health policy makers need to engage with urban Indigenous leaders to take decolonizing steps that include applying understanding of the specific ways multiple and complex historical legacies intersect and impact urban Indigenous peoples’ health and access to health care. These include consequences for data collection problems, mistrust, the perpetuation of racist ideologies of blame, and misrecognition of the nature of the barriers faced by urban Indigenous populations. Systemic violence against Indigenous women and gender-diverse people in urban areas has been woefully neglected.

• Urban Indigenous organizations must be supported in administering public health services themselves, be more fully engaged in critical care settings and in their networks of cooperation and knowledge sharing.
• Public health messaging should recognize the diversity of Indigenous peoples in urban areas, as well as the intersectionality of race, gender, and other structures of oppression, and ensure that the appropriate community authorities are supported in their approaches to reaching their constituents.
• Shelters are urgently needed for those fleeing domestic violence escalated by the pandemic.
• Health data collection policy is required that meets the needs identified by these communities themselves, and the resources to address these needs.

Lack of policy in both countries dismisses and undercounts the COVID-19 human consequences for Indigenous peoples, especially those residing in urban communities. Comprehensive data will be key in understanding how COVID-19 is affecting Indigenous people, directing resources to meet their needs, and measuring response outcomes and relief efforts. Without meaningful data, government response is often delayed or not given. Long-standing organizations like the NAFC in Canada and the Urban Indian Health Institute in the United States are critical to improving health data because they belong to the coalitions of urban-based agencies that serve Indigenous peoples.

Second, while it has been stated countless ways by many parties, federal assistance for the delivery of health care, including mental health, for and by urban Indigenous peoples needs to be substantially increased to match the needs of populations in urban areas and in coordination with urgently required improvements in housing, education, food security, and Elder care.

• There needs to be a concerted effort to develop with urban Indigenous leadership long-term economic security planning.
• Urban Indigenous organizations need more resources and clear-cut federal policies to support the services they provide.
• The competition-based approach to funding that pits urban against rural/reservation communities and among themselves needs to end.
• The pandemic highlighted the need for increased affordable access to basic personal computer equipment and the internet, often assumed to be a problem only for remote rural communities.
• Mediation of the vulnerabilities of Indigenous children to long-term impacts of COVID-19 needs to be attended to under the supported leadership of urban Indigenous communities, including family and kinship disruption, stunting access to education, and increased risks of homelessness.

Finally, safeguards to protect basic rights to practice cultural activities and access spiritual well-being need to be considered as important to overall health maintenance as sustenance and shelter. Women’s roles as caretakers, and notably the positionality of midwives, provide insights into intimate forms of both suffering and coping, and the culture-based pathways to community health.

• Urban Indigenous community leadership need to be supported in their emphasis on delivering cultural and spiritual services.
• In addition to mediating against the discontinuity of cultural services, attention to social and health care service provider wellness should be integrated into future pandemic planning.

Urban Indigenous program staff adapted quickly to needs of their communities and their innovative approach and assistance are accepted and appreciated. Indigenous urban community resiliency is rooted in the spirit of relationships, a form of strong intertribal kinship that makes every patient or client a kin or family member. Familiar agencies and other resources are successful because the cadres of these Indigenous first responders are trusted and committed. They are quick to change their role in some instances by delivering food and medicine, or by setting up alternative ways to find help. Where they cannot deliver medical care, they inform their constituents about keeping safe, where to go for testing, assist with contact tracing, and should continue leadership in vaccination roll-out. Knowing that viral transmission depends on human behavior embedded in structural inequities and social relations is as important as understanding virology, when preventing or slowing epidemics (Editorial, BJSP, 2020). To prepare a community to respond successfully to a life-threatening situation requires the messengers to be familiar, trusted, reliable, and a source of cultural safety.
